# Supplementary material for: Learning-oriented motivation: Examining the impact of teaching practices with motivational potential
Source: PLoS One. 2024 Feb 23;19(2):e0297877. doi: 10.1371/journal.pone.0297877 (PMC10890759; doi:10.1371/journal.pone.0297877)
Supplement: S1 Appendix — (DOCX) [file pone.0297877.s001.docx]

**Items from the inventory to measure the Motivational Potential of motivational teaching practices (Original version in Spanish)**

**Potencial Motivador de las prácticas motivadoras docentes**

Jorge Valenzuela, Jorge Miranda-Ossandón, Carla Muñoz , Andrea Precht, & Milenko Del Valle

**Qué tan a menudo los profesores realizan estas prácticas ( 0=nunca - 5 siempre)**

| 1. Proponen recompensas (puntos, décimas adicionales) |
| --- |
| 1. Proponen sanciones (descontar puntos, reprobación de la asignatura) |
| 1. Muestran la utilidad de los contenidos que enseñan |
| 1. Destaca la importancia de aprender los contenidos del curso para el desarrollo profesional |
| 1. Suscita o fortalece el interés por el contenido de la asignatura |
| 1. Desafía a los alumnos a aprender mostrándoles que son capaces |
| 1. Demuestra empatía, cercanía o comprensión con los alumnos |
| 1. Genera un clima en el aula que te hace sentir seguro/a para participar de la clase |
| 1. El profesor propone actividades para que usted trabaje de manera autónoma |

**Las siguientes prácticas de los docentes me generan deseos de aprender en la carrera**
(0=Totalmente en desacuerdo 5=totalmente de acuerdo)

| 1. Proponen recompensas (puntos, décimas adicionales) |
| --- |
| 1. Proponen sanciones (descontar puntos, reprobación de la asignatura) |
| 1. Muestran la utilidad de los contenidos que enseñan |
| 1. Destaca la importancia de aprender los contenidos del curso para el desarrollo profesional |
| 1. Suscita o fortalece el interés por el contenido de la asignatura |
| 1. Desafía a los alumnos a aprender mostrándoles que son capaces |
| 1. Demuestra empatía, cercanía o comprensión con los alumnos |
| 1. Genera un clima en en el aula que te hace sentir seguro/a para participar de la clase |
| 1. El profesor propone actividades para que usted trabaje de manera autónoma |
