# Supplementary material for: Learning-oriented motivation: Examining the impact of teaching practices with motivational potential
Source: PLoS One. 2024 Feb 23;19(2):e0297877. doi: 10.1371/journal.pone.0297877 (PMC10890759; doi:10.1371/journal.pone.0297877)
Supplement: Attachment — Submitted filename: response to reviewers.docx [file pone.0297877.s002.docx]

**Response to reviewers**

We want to thank the reviewers for their comments and suggestions for improving the manuscript. We attach the details of the changes made to consider all the reviewers' suggestions.

Some other changes were made, such as including references or changes in writing.

The link to access the database is also added, and the original questionnaire used to assess the motivational potential of teaching practices is annexed.

**Editor**

1. Please ensure that your manuscript meets PLOS ONE's style requirements, including those for file naming. The PLOS ONE style templates

We have revised the manuscript and corrected from the PLOSONE standards.

2. 2. We note that the grant information you provided in the ‘Funding Information’ and ‘Financial Disclosure’ sections do not match. When you resubmit, please ensure that you provide the correct grant numbers for the awards you received for your study in the ‘Funding Information’ section.

The funding sources have been corrected by specifying the grant number and the researcher receiving the grant.

3. In your Data Availability statement, you have not specified where the minimal data set underlying the results described in your manuscript can be found.

The data underlying the results of this article are available at: <https://doi.org/10.5281/zenodo.10427851>

Reviewer 1

1. In the first paragraph of introduction, the authors bring in the term motivational orientation. Please provide a definition of motivational orientation.

A definition of motivational orientation is incorporated and made explicit in the first paragraph of the introduction.

2. Regarding the introduction, it would be beneficial to expand on the theoretical frameworks that form the basis of the study. Consider providing a brief explanation of each and their contribution to understanding the motivation to learn.

The contribution that theories make to the understanding of motivation to learn is made explicit in the introduction.

3. At the end of introduction, the authors mention that current literature shows the benefits of integrating different theories. Please explain in more detail what those benefits are.

It makes explicit the meaning and benefits of integrating different theories. (See rows 92 et seq.)

4. The same aspect can be covered in more detail in discussion (rows 259-261). As integrating theories is one of the strengths of the study, it would be beneficial to provide more information about the specific findings of the cited studies and their relevance to the current research.

This aspect is covered in discussion (see rows 298 et seq.)

5. In the methods section, I find it necessary to clarify how the questionnaire was developed. Were the items used to measure the motivational potential of practices based on previously used instruments? I would encourage authors to make the administered questionnaire available. Also, please include information about the verification of the psychometric characteristics of the scales (e.g., in an additional file).

The origin of the instrument is explained, and the questionnaire is annexed. See appendix S1

a. It would be helpful to use the same terminology throughout the text (e.g., utility vs usefulness). Do learning orientation (row 18) and motivational orientation have the same meaning?

To avoid confusion, the terminology "utility" is homogenized throughout the text.

In the case of "learning orientation" and "motivational orientation, the expression is homogenized as "orientation towards learning" throughout the text, and it is made explicit that this is a specific case of motivational orientation).

b. Some of the sentences could be better worded for clear understanding (e.g., rows 22-24 “While the effects…”; rows 95-98 “In this context…”).

B. About rows 22-24 the paragraph is rewrite:

(see rows 27 et seq.)

*Although the impact on motivational orientation toward learning may vary slightly across different fields of study, the primary predictors consistently are practices that emphasize importance and foster autonomy. The effect of utility-focused motivational practices is observed only within the predictive model for the group of health students*.

c. In discussion, the paragraph in rows 223-230 lists the three significant practices twice. Please consider removing the repetition.

c. the text (rows 223-230) was reformulated: (see rows 260 et seq.)

*Only three of the nine practices examined demonstrated a statistically significant influence on the motivational orientation toward learning. Specifically, motivational practices that emphasized the importance, autonomy, and utility of learning were found to have a significant impact. However, the utility variable exhibited significance solely within the health students' group and with a marginal contribution. Consequently, there is a discernible divergence in the effects of these variables between the two groups. Notably, practices centered around reward, punishment, challenge and self-efficacy, emotional support, and creating a safe classroom environment did not exhibit a statistically significant contribution to the model.*

d. All in all, the language of the manuscript is good. However, there are some sentences that could be improved to make the text easier to follow. I would recommend asking a native English speaker to check the wording of the manuscript.

The suggestion was welcome

**Reviewer 2**

1.The sample consists of Chilean university students. At this point, it is recommended to declare the size of the population, differentiated by gender, the different types of universities that exist in Chile. Characterize the population of university students.

A summary of the university education system in Chile is added in the "participants" section.

The fact that the Chilean educational system includes both public and private universities is made explicit, and the configuration of the participants is detailed.

(See Participants section)

2. Characterize the sample, indicating the percentage it represents of the population, for each type of university, regions, etc. This is relevant to have clarity on who your sample is representing.

A more precise characterization of the participants was carried out.

3. The value of the Determination Coefficient is striking, as it does not exceed 25%. It should be explained that there is more than 75% of the variability of the dependent variable that is not being explained by the variables considered in the study and conduct a discussion on the potential of these findings, which are rather limited.

A commentary focused on this aspect (the effect size observed in this study) is included in the discussion section, comparing it with the results of Hattie's meta-analysis. (See rows 307 et seq).

4. In Table 1, it is suggested to remove the column of sample sizes for health and education and refer to them in the text.

The column of sample sizes for health and education were removed. The number of participants is reported in footnote
